# Analyze the Wear Mechanism of the Longwall Shearer Haulage System

**DOI:** 10.3390/ma16083090

**Published:** 2023-04-13

**Authors:** Krzysztof Jaśkowiec, Zenon Pirowski, Mirosław Głowacki, Magdalena Bisztyga-Szklarz, Adam Bitka, Marcin Małysza, Dorota Wilk-Kołodziejczyk

**Affiliations:** 1 Łukasiewicz Research Network—Krakow Institute of Technology, Zakopiańska 73, 30-418 Kraków, Poland; 2 Faculty of Metals Engineering and Industrial Computer Science, AGH University of Science and Technology in Krakow, al. Mickiewicza 30, 30-059 Kraków, Poland; 3 Faculty of Natural Sciences, Jan Kochanowski University of Kielce, ul. Żeromskiego, 25-369 Kielce, Poland

**Keywords:** wear, adhesive wear, carburizing, 20H2N4A, haulage system, longwall shearer

## Abstract

The wear characteristics and related mechanisms of the Longwall Shearer Haulage System were investigated. Wear is one of the main reasons for failures and downtimes. This knowledge can help solve engineering problems. The research was carried out at a laboratory station and a test stand. The publication presents the results of tribological tests carried out in laboratory conditions. The aim research was to select the alloy intended for casting the toothed segments of the haulage system. The track wheel was made by the forging method using steel 20H2N4A. Haulage System was tested on the ground using a longwall shearer. Selected toothed segments were subjected to tests on this stand. The cooperation of the track wheel and toothed segments in the tootbar were analyzed by a 3D scanner. Debris chemical composition was also appointed, as well as mass loss of toothed segments. The developed solution toothed segment an increase in the service life of the track wheel in real conditions. The results of the research also contribute to reducing the operating costs of the mining process.

## 1. Introduction

In mining, a large amount of energy loss results from friction and wear of working elements. It is estimated that 38% of the energy consumed in mines is related to friction. Friction also affects the efficiency of the entire mining process. Longwall shearers are an example of a device where friction is a significant factor of wear. The wear may concern the teeth of the drive wheel itself, as well as other elements, e.g., the entire haulage system of a longwall shearer [1]. Eicotrack is a haulage system very often used in mines, although it is not adapted to modern requirements. Since the 90s of the last century, longwall shearer have been equipped with automated systems increasing safety and mining capacity. As a result, the shearers’ development is, among others, an increase in their pulling force. In connection with the rigid structure of the haulage system, this increases the tribological wear. In this case, wear can cause breakdowns, downtime and losses during mining works [2,3].

**Figure 1:**
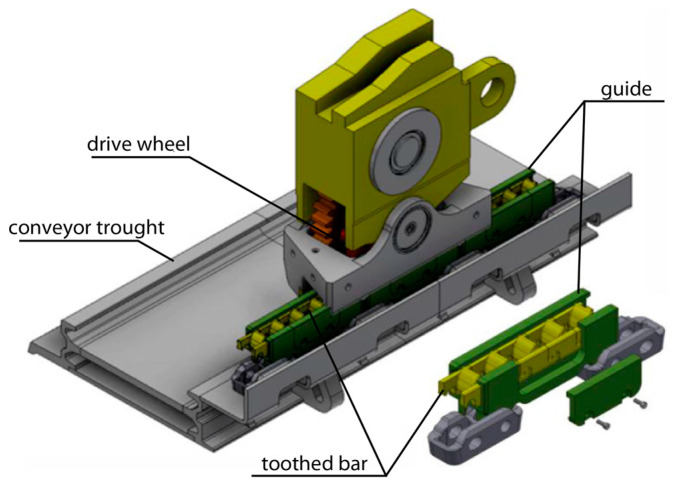
Haulage system 3D model [13].

The specific working conditions of the haulage system toothed segments in cooperation with the wheel of the longwall shearer give an extra requirement for the materials used. Alloys intended for the production of these parts should cut the degree of wear of the shearer’s longwall drive wheel [4]. Figure 1 shows the method of cooperation between the wheel and the newly developed haulage system. On the conveyor through there are guide elements in which individual toothed bar segments are placed. The drive wheel cooperates with these segments, causing the movement of the shearer. Replacing this wheel during mining processes is complicated. This operation is a time-consuming operation that causes significant production downtime. One of the ways to increase service life and safety is by changing the design. Another way is the appropriate change of the materials used. These solutions are proven paths in the mining industry [5]. The material used for the drive wheels of the longwall shearer is carburized nickel-chromium steel 20H2N4A. This grade of steel is designed to work with heavy loads. It can be carburized and subjected to heat treatment, which consists of quenching and tempering. After this process, a carburized surface with a hardness close to 60 HRC is obtained. The carburized layer can reach a few millimeters deep into the material, depending on the method used and the parameters of the heat treatment process. The layer is resistant to wear and rolling contact fatigue. Although many modern materials have appeared, this grade still remains competitive [6,7,8]. During the longwall shearer work, wheel and toothed segments cooperate with each other. These elements constitute a friction pair in the operation process. It seems that hardness may be one of the parameters of the choice of materials intended for rack segment production. The hardness of a material is an important factor in proving its resistance to wear. However, hardness does not have to be the decisive factor. One of the most important goals of the work carried out is to get the possible lowest wear of the drive wheel together with the longest service life of the toothed segments [9,10,11].

Due to the characteristics of the deposit, the direction of the path of the conveyor troughs is not straight, and it can bend both vertically and horizontally. This affects the haulage system, and in conjunction with the rigid attachment of some elements (e.g., toothed bars) of this system, the position of the shearer’s drive wheel relative to the axis of the tooth segments may change. In the case of changes in the direction of the track in the vertical plane, the permissible contact stresses may be significantly exceeded. They may show so-called tooth edges, which significantly accelerate the process of wheel wear. Another mechanism occurring in the process of wear of the friction pair between the drive wheel and the tooth segments is slip. The mentioned mechanisms accelerate the process of wear of the wheel and toothed segments, necessitating the replacement of the drive wheel. These effects can be minimized by changing the design elements of the haulage system. On the other hand, focusing on specific parameters, e.g., by reducing contact stresses, may cause more intensive wear of wheels and toothed segments compared to the old design. Regardless of the design of the haulage system, as in each friction pair, various wear mechanisms may occur [12,13,14,15].

In coal mining, machinery and equipment are exposed to aggressive environments and loads. Working surfaces can wear due to various wear mechanisms. One of the most common wear mechanisms in mining is abrasion, and a combination of plastic deformation, abrasion and oxidative wear can also be encountered. Other wear mechanisms that can occur in mining are adhesive wear and fatigue. In a friction pair, the wear mechanisms may depend on several conditions, one of which is the type of cooperating materials [16,17,18].

Austempered ductile iron (ADI) has good mechanical properties and wear resistance. In ADI, the wear resistance of the surface layer can be increased by a transformation of the austenite into martensite. The transformation process is possible under load [19,20]. The second group of analyzed alloys was several grades of cast steel, including high manganese cast steel which ensures high resistance to abrasive wear. These alloys have many applications in cement plants, transport and mining, for example, a roller ore crusher. Hadfield cast steel with an Mn content of 10–14% is used there due to the well-known excellent abrasive resistance. A number of phenomena, such as twin-twin hardening, dislocation hardening, and dynamic strain gain, among others, influence this property. At the same time, these alloys show good mechanical and plastic properties [21,22]. Other tested cast steel grades contained elements such as Cr, Ni, Mo, and V in their chemical composition. Some of these elements contribute to the formation of carbides which play an important role in the wear process. The heat treatment also affects the obtained microstructure and, thus, the properties [9,23]. This heat treatment consists of quenching and tempering (Q&T), a process that is often used in the production of steel-cast components. In the quenching process, alloying elements might be required if the martensitic structure is to be obtained in the entire volume in a diffusionless transition process. After the quenching process, the microstructure of cast steel gives high hardness and strength, but, at the same time, low plasticity and fracture resistance are achieved due to hardening stresses and high dislocation density. Performing a tempering procedure can change this. When the tempering temperature does not exceed 250 °C, the α’ phase or the carbon supersaturated solution is transformed into tempered martensite α’’ due to carbon diffusion and precipitation of Fe_2_C–Fe_2,4_C type carbides, or Fe_3_C if the tempering temperature is higher. During the tempering process, bainite may also be formed as a result of the transformation of the residual austenite [24,25]. The final properties of toughened steels depend on the microstructure that evolves during the Q&T process. In the design of the heat treatment process, we can use computer analysis. However, in practice, the Q&T process is often designed using empirical methods. Even in this case, differences in both the alloy composition and the geometry may result in a much lower ability to predict the properties of the designed part [26]. After the Q&T process, a mixed structure may be obtained, which consists of martensite, residual austenite, bainite and perlite. This list does not exhaust all the components of the structure that can be obtained in the Q&T process in a steel casting. This list is presented in descending order due to the wear resistance. The relationship between microstructure and wear resistance has not been fully explained so far. It is generally accepted that the main factors influencing the wear of martensitic steels are hardness and ductility. Li also draws attention to carbides and their morphology. One of the conclusions obtained by Trevisiol is the influence on the wear resistance of factors such as the morphology of martensite or the two-phase structure of the alloy [27,28,29,30,31].

The literature review of longwall shearer haulage systems focuses mainly on the drive system modeling, and there is much less information about the tribological properties of the friction pairs of materials used in cooperating elements. There is not enough data in the literature describing alloy the work of the entire friction pair where the carburized steel for drive wheel longwall shearer is one of the materials [32,33,34].

## 2. Materials and Methods

### 2.1. Materials

The samples of chosen grades of cast steel and ADI cast iron were taken from cast ingots obtained from melts conducted in an open induction furnace in a crucible with a capacity of 100 kg with an inert lining based on Al_2_O_3_. Chemical analysis of the investigated alloys was made by emission spectrometry using the GNR MINILAB 300 device. The obtained results are summarized in Table 1.

Normalizing heat treatment of cast steels was carried out in an electric resistance furnace POK73.1. The austenitization step before quenching was carried out in an electric muffle furnace Multitherm N41/M under an argon protective atmosphere. The tempering procedure was also carried out in the same Multitherm N41/M furnace. In this process, for a temperature of 600 °C, the treatment was carried out under an argon atmosphere, and for a lower temperature, in an air conditioner. Heat treatment parameters of tested alloys are shown in Table 2.

### 2.2. Microstructural Analysis

The substrate interface and microstructure were studied by scanning electron microscopy (SEM) and energy dispersive x-ray spectroscopy (EDS) using Scios, FEG and FEI. The metallographic examination was performed by optical microscope (Axio (New York, NY, USA) Observer. Z1m). Keyence (Osaka, Japan) VHX-700F optical microscope was utilized to characterize the 3D surface topography.

### 2.3. Mechanical Testing

The samples were cut out from the toothed segment castings. Microhardness, hardness and tensile tests were conducted to determine the mechanical properties of the obtained specimens. The static tensile tests were conducted by employing Instron (Norwood, MA, USA) 8800M hydraulic testing machines, whereas the hardness tests used Zwick/Roell typ BHO25.HZHU.3000 accordances with the PN-EN ISO 6892-1: 2016-09 and PN-EN ISO 6506-1: 2014 standards, respectively.

Microhardness distribution measurements were performed on an Anton Paar (Graz, Austria) microhardness device. Hardness measurements were performed using the HV hardness scale under a load of 1 N. The measurement was made on the carburized surface into the material.

### 2.4. Wear Testing

**Figure 2:**
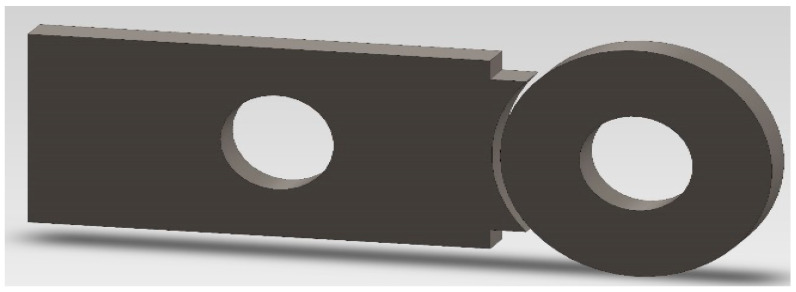
Sample and counter-sample used in the Amsler-A135 Machine.

An abrasion test in a macro scale was carried out, which enables the measurement of wear resistance of the surface areas of the material. The Amsler and the ‘pin-on-disc’ tribological systems weres used. Initial wear tests were carried out on an Amsler-A135 (Amsler, Schaffhausen, Switzerland) machine. In a dry friction condition test, the force at the level of 577 N was applied on the sample against the counter-sample at the rotational speed of the shaft equal to 200 rpm (0.42 ms^−1^), on which the counter (sample) was mounted (Figure 2) The samples were taken from heat treated castings made from alloys selected for testing. The counter-samples were cut out from the drive wheel (20H2N4A). Measurements were made after 2, 4, 6 and 8h.

**Figure 3:**
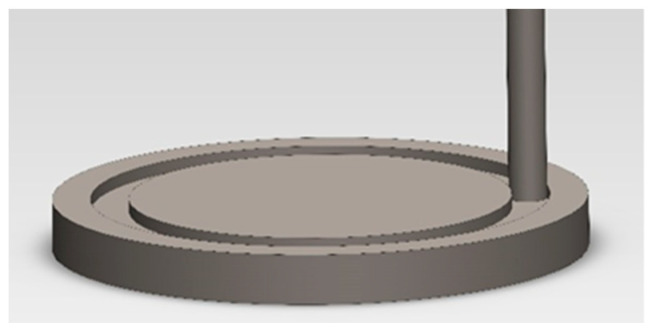
Scheme of the pin-on-disc method.

**Figure 4:**
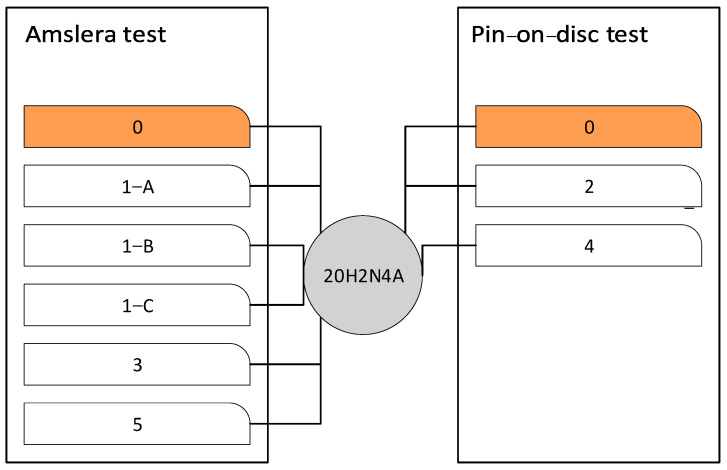
The tested friction pairs and the platforms used to assess the wear resistance.

A pin-on-disc type tribometer model TR-20 was used to wear activity under dry sliding conditions. In these tests, the sample in the shape of a pin is loaded with a constant force against the rotating disk (the counter-sample), which results in the effect of friction. The measure of resistance to friction wear is the loss of sample mass as a result of the test. Before and after running the test, the weight of pins was evaluated using an electronic microbalance with high accuracy (±0.1 mg). Other indicators of the wear process are the kinetics of changes in the friction coefficient and kinetics of the total wear of the friction pair. A pin-on-disk (POD) tribo-testing machine was used to measure the wear and friction coefficient on the influence of the samples. The pins, measuring 3 mm in diameter and 25 mm in length, were made of a drive wheel. The discs measured 50 mm in diameter and 5 mm in thickness and were made of materials marked with the symbols 0, 2 and 4. Contact at the sliding interfaces was under a force of 45 N, as shown in Figure 3. The sliding speed was 0.25 m·s^−1^ for the tribological experiments, and the sliding time was 23 h. The friction distance was 22,780 m. Tests were carried out under ambient conditions. The material pairs and platform used to perform the tests are shown in Figure 4.

### 2.5. Longwall Shearer Test Stand

The longwall shearers operation was carried out in the great outdoors. The haulage system and other elements necessary during the operation of the device were prepared. A stand was used to simulate the load which may occur during the longwall shearer operation in in-situ (underground) conditions. It was assumed that the largest force transmitted by a tooth would be 562 kN. The angle of rotation of the toothed bars (guide) in relation to each other is a maximum of ±0.3 degrees [27]. During the test, the longwall shearer was driven in one direction under load and back to an idle state. The haulage system route started with a straight section, then the haulage system climbed the bend in the horizontal plane, and then the vertical bend was simulated. A total of 47 runs of the shearer were performed.

After the operation tests, the toothed segment wear measurements were carried out using the Atos III 3D optical scanner (Atos, Bezons, France). To measure the operational dimensional changes, solids of individual castings scanned before and after the tests were matched with the best-fit method. The weight loss of selected toothed segments was also determined. A Rawag WLC30/K/F (Radom, Poland) scale with a most measuring weight of 30 kg and an accuracy of 0.5 g was used to measure the weight of castings before and after the operation tests.

## 3. Results and Discussion

### 3.1. Mechanical Properties

Table 3 presents the mechanical properties obtained from the tested alloys. The results obtained for these alloys differ significantly. It must be noted properties of alloys to be used in this research needed to meet the requirements of the haulage system design, e.g., elongation (A) should be greater than 8%. Unfortunately, alloys 1–1, 1–2, 3 and 4 do not meet the required value. Nevertheless, it was decided that further tests would be carried out for all materials due to possible changes in the surface layer of the samples during the wear test, especially hardness.

**Figure 5:**
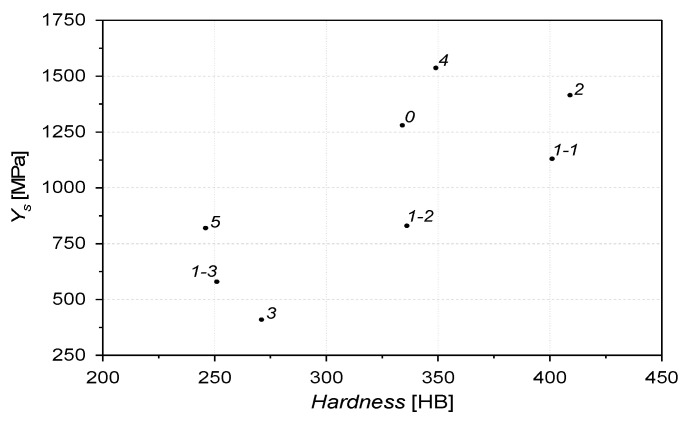
Correlation between yield strength and hardness of tested alloys.

The analysis of Table 3 and Figure 5 shows the evident division of the tested alloys into three groups: I—alloys with a hardness below 270 HB, II—alloys with a hardness oscillating around 340 HB, and III—alloys with a hardness above 400 HB. The hardness of samples 1–3, 3 and 5 seems to be too low compared to the desired value. However, referring to the hardness obtained after the changes of the surface of some materials during the operation process is more adequate. During the exploitation process, hardness of the surface layer may become much higher. In the case of sample 3, the hardness achievable under pressure exceeds 500 HB. In the case of alloys 1–3, residual austenite may transform into martensite if the load limits are exceeded [35,36,37]. The yield strength may also be a parameter influencing the wear resistance. Zambrano examined three steel grades of the same hardness but different values of yield strength. He showed a relationship between yield strength and wear mechanisms such as micro-cutting and grooving. He also confirmed the approximate relationship between wear and yield strength [38].

### 3.2. Microstructure of 20H2N4A

**Figure 6:**
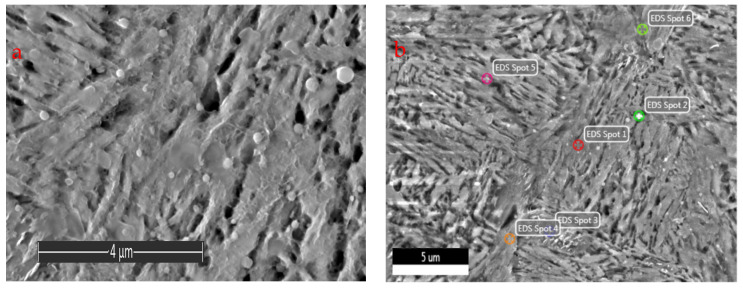
(**a**) SEM micrograph; and (**b**) EDS analysis points showing microstructure of the 20H2N4A.

SEM image and EDS analysis points of the 20H2N4A steel subjected to carburization are shown in Figure 6a,b, respectively. The sample for microstructure examination was collected from the surface layer of the driving wheel. The matrix is tempered martensite. The presence of spheroidal particles of carbides can be noticed. Literature data state that the carbides of this shape are complex carbides of the (FeCrMn)C type. Their presence may be related to the fact that they did not dissolve in the matrix during the austenitizing treatment. These precipitates can increase the abrasion resistance of the carburized layer [23,39]. The chemical composition analysis of the core and the surface of the drive wheel are given in Table 1., The average carbon content on the surface is 0.59%, while in the core, 0.17%. The EDS point analysis (Figure 6b) has shown fluctuations of chromium and nickel, as presented in Table 4. The presence of spheroidal precipitates on the area marked as “Spot 2” (Figure 6b), there is a noticeable increase of carbon and chromium concentration, which was not observed the case in the other studied areas. These carbides can inhibit the growth of austenite grains, affecting the properties of the obtained carburized layer [40].

### 3.3. Distribution of the Microhardness of the Carburized Layer of the 20H2N4A Alloy

**Figure 7:**
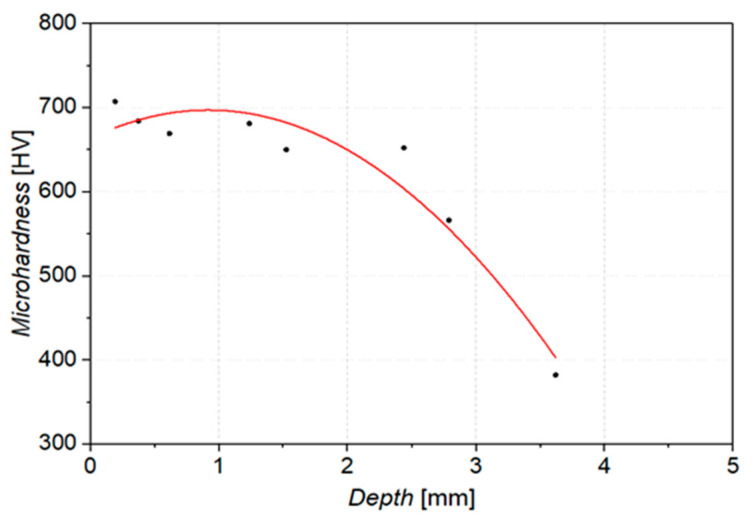
Dependence of microhardness as a function of depth.

Figure 7 shows the distribution of hardness as a function of depth. The measurement was carried out over a distance of 4 mm. The depth dependence of hardness decreases with increasing the distance from the surface. The hardness of 550 HV is achieved at a depth of 2.90 mm. Except for the carburized layer, the measured hardness was 380 HV up to 14 mm in depth. With such a thickness of the carburized layer, it can be expected that samples (pins) made from 20H2N4A steel will cooperate with the counter-sample only by contacting with the carburized layer throughout the tribological test [41].

### 3.4. Wear Behavior

#### 3.4.1. Amsler Test

**Figure 8:**
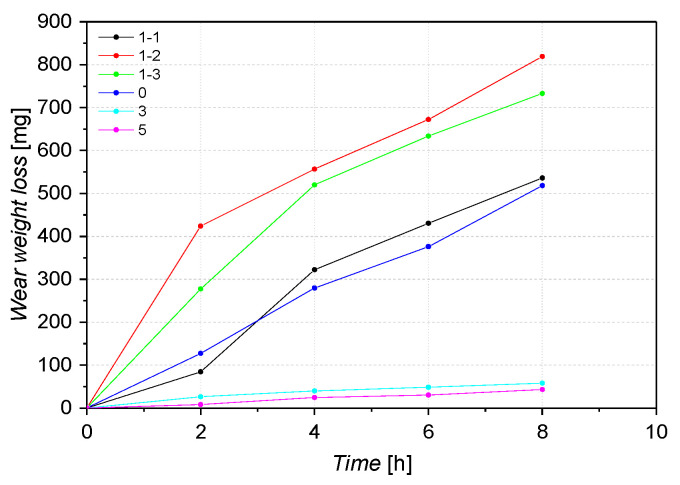
Weight loss of samples as a function of time.

Figure 8 shows the weight loss of the six alloys tested in pairs with 20H2N4A steel, where this steel was a counter-sample. Alloys marked 3 and 5 showed similar wear resistance under these conditions. The weight loss of sample 5 was the lowest and amounted to 42.9 mg/8h. For alloys 0, 3 and 5, the weight loss increased almost linearly throughout the experiment. For samples 1–2 and 1–3, the weight loss over time can be divided into two stages. In the first hours of the experiment (about 2 h for specimen 1–2, quenched at T = 330 °C and 4 h for specimen 1–3, quenched at 375 °C), the loss was higher, and then it slowed down. Sample 1–1 (quenched at T = 270 °C) showed the highest wear resistance compared to the other ADI cast iron samples tested. Other researchers [42] report that there is a significant difference in the wear resistance even in the ADI cast iron quenched at 320 °C and 340 °C. This may be related to the differences in the participation of retained austenite in the matrix. The better wear resistance of sample 1–1 quenched at 270 °C is consistent with the literature data too. It seems that during tests done in this research, the transformation of residual austenite into martensite on the surface layer of samples 1–2 or 1–3 did not occur [43,44]. In the case of sample 0, significant weight loss was observed. After eight hours, the wear was almost 10 times greater than that of alloy 5 (Figure 8). Martensitic cast steels, including cast steel 0, and in comparison to sample 5, do not have the addition of V and also have a lower Mo content. Cast steel 0 obtains its tribological properties not only due to the surface hardness but the distribution and size of the carbides may also be important [45]. As shown in Figure 5, cast iron 1–1 has the highest hardness of the ADI cast irons tested. Moreover, sample 1–1 had an approximately 50 HB harder than the alloy from which sample 0 was made. Despite this, an almost identical wear value was obtained for the tested samples.

**Figure 9:**
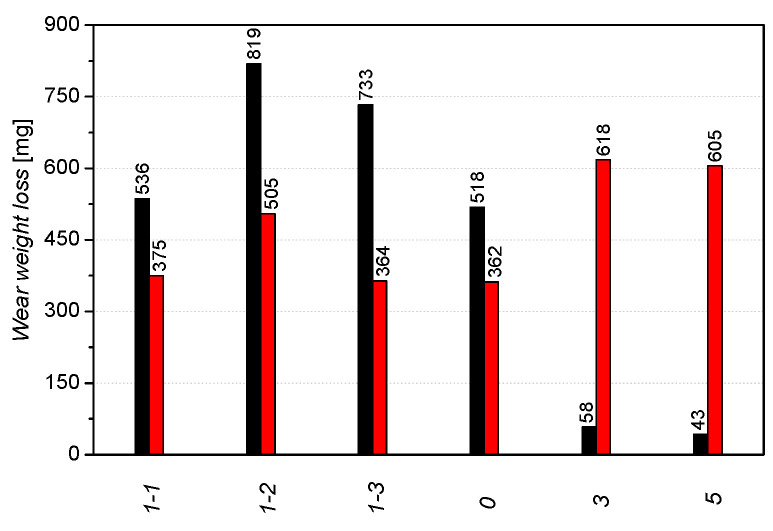
Results of the Amsler test of the sample (black) and counter-sample (20H2N4A, red) after 8 h.

Figure 9 shows the weight loss of the tested friction pairs system after 8 h of testing on the Amsler stand. After tests, a significant differentiation of the obtained results was found. The most favorable wear resistance ratio in the sample/counter-sample set was demonstrated by the 1-1/20H2N4A and 0/20H2N4A pairs. The weight loss ratio of the sample to the counter-sample was 536/375 and 518/362.

The friction coefficient for the pair with ADI/20H2N4A marked with the symbol 1–3 was determined at the value of 0.21. For the remaining alloys, this coefficient was higher and was equal to 0.28–0.29.

The temperature of friction pair samples during the tests was also measured. The obtained temperatures ranged between 90 and 99 °C. The highest temperatures were obtained for the friction pairs with alloys 3 and 5. Among the ADI alloys, sample 1–1 showed the highest pair heating temperature, equal to 94 °C. Despite similar results obtained during the wear test for samples 1–1 and 0, the measured temperature was different for these alloys. For alloy 0, the lowest temperature was recorded among all the alloys tested on the Amsler stand (90 °C).

Most researchers assume that almost all work done by friction manifests as heat generated in a thin contact layer [46]. Both the lowest temperature recorded as well as the friction test results show that pair 0/20H2N4A is the most favorable from the wear resistance point of view.

#### 3.4.2. Pin-on-Disc Test

**Figure 10:**
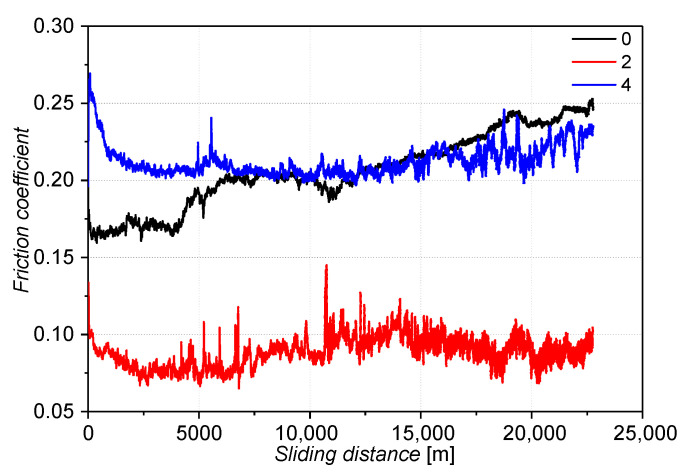
Coefficient of friction of the tested alloys by the pin-on-disc method.

Figure 10 presents a diagram of the friction coefficient for the samples tested using a tribological pin-on-disc method. For these tests, it should be taken the potential change of the friction coefficient in the initial phase of the test. This change may be the result of changes in stress on the surface of the worn sample [47]. For samples 2 and 4, the friction coefficient decreased at the beginning of the test. After about 3000 m of sliding distance, the friction coefficient stabilized at the value 0.21 for sample 4 and 0.075 for sample 2. The low friction coefficient for sample 2 may be related to different wear mechanisms and high yield points. In the composition of the sample 2 alloy, there is boron. This element increases the resistance to wear resistance, as well as reduces the coefficient of friction [48,49]. The coefficient of friction for sample 0 increased along with the sliding distance from the group of tested materials.

**Figure 11:**
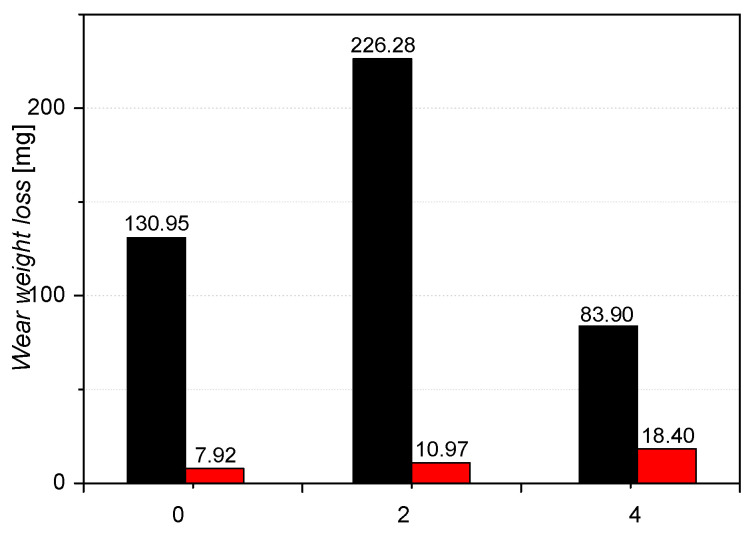
Weight loss of the pin (red) and disc (black) for the tests carried out on the pin-on-disc stand.

**Figure 12:**
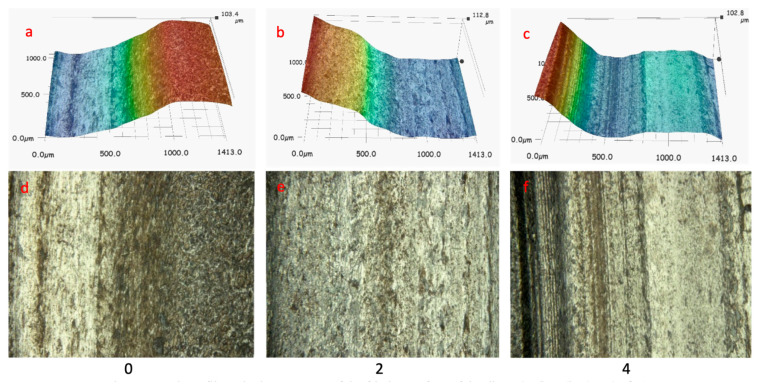
Depth profile and microstructure of the friction surface of the discs: (**a**,**d**) 0; (**b**,**e**) 2; (**c**,**f**) 4.

The mass losses of the pins results are shown in Figure 11. Three replicate tests were performed for the disc and pin. For the tested discs, the standard deviation was equal: “0”—28.5; “2”—38.4; “4”—2.2. The standard deviation for tested pins was equal: “0”—0.81; “2”—1.03; “4”—0.98. The greatest loss of mass was recorded for sample 4. Samples 4 and 5 (sample 5 tested on an Amsler stand) showed the lowest weight loss during the tests while affecting the pin the most (20H2N4A). Both aforementioned samples contain vanadium, which tends to form temperature-stable carbides VC. Mo, which forms carbides (Mo_2_C) and sulphides, may behave similarly. In the case of sample 4, the highest value of yield strength (YS) was obtained among the tested group of alloys (Table 3). YS can have an effect on the results of sample 4 during the wear-resistance test [50]. The pin wear results for alloys 0 and 2 correlate with the obtained hardness, which was respectively 341 HB and 409 HB. For these two alloys, the difference in values of hardness results from the content of Mo and Cr and differences in the heat treatment process. However, higher hardness has not inflicted less weight loss on the disk made from alloy 2, which was the largest among the investigated alloys during pin-on-disc testing. As was the case with the tests carried out at the Amsler stand, also pin-on-disc method shows that the friction pair 0/20H2N4A exhibit the best resistance to wear. The weight loss of pin (for pair 0/20H2N4A) is 28% lower compared to pair 2/20H2N4A. In the case of alloy 4, the wear of the pin is more than twice as high. Figure 12 shows selected fragments of the disc wear profiles and their microstructure for samples 0, 2, and 4. During the 23 h of the test, wear products may accumulate locally, affecting the wear of the tested friction pair. The smallest changes in profile are shown for sample 4. The surface of both the disk and pin were wearing quite evenly over the entire contact plane. In the case of sample 2, and especially sample 0, there are large differences in the wear of the pin and disk areas. Wear mechanisms may depend on the distribution of wear debris in the sliding track when the adhesive is significant [51].

**Figure 13:**
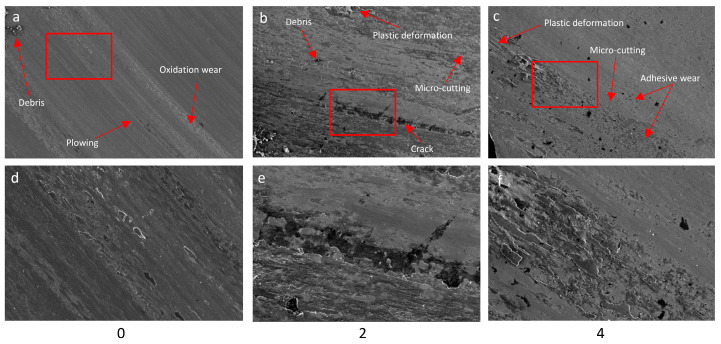
SEM image of worn surface pin after test: (**a**,**d**) 0; (**b**,**e**) 2; (**c**,**f**) 4.

Figure 13 shows the working surfaces of the pins after the pin-on-disc tests. In the case of alloy 0, areas covered by an oxidized layer can be found on the pin. Oxidative wear is caused by the heat generated between the friction pair during the friction process. The metal surface reacts with the oxygen in the air to form oxides. Oxides produced during movement can lose integrity on the surface, creating wear debris. This debris will get compacted together when sliding, causing less wear [52]. We can see plowings on the pin surface for pair 0/20H2N4A, and this abrasion mechanism is important for these two-body contact. Scuff marks in micro-cutting are visible, as well as an area with accumulated debris such as loose oxides. Adhesive wear is noticeable, and this wear mechanism adds more hard debris.

The oxidation degree of the pin cooperating with the disc made of alloy 2 is much lower than that of alloy 0. On the surface of the pin, a continuous foreign material layer was deposited. This layer is permanently bonded to the pin surface, a phenomenon characteristic of adhesive wear. This may explain the value of the friction coefficient obtained for this friction pair (Figure 10) [47]. Considerable wear of the disk (Figure 11) also confirms this supposition. Debris is not bound to the surface, causing micro-cracking and plastic deformation. The occurrence of cracks on the sample surface is related to fatigue wear. In the pin, there are noticeable grooves resulting from the rolling of fragments of abrasion products between the rubbing surfaces (3 body wear) [53]. The third body may play a role in the lower friction coefficient, especially when both abrasive wear and adhesive wear exist together. When the adhesion tendency is slight, the third body influence on friction coefficient and mass loss is reduced. For the friction pair whit disc 2, the role of the third body in the surface damage could be significant [51,54].

In the case of a pin cooperating with a disc made of alloy 4, adhesive wear of the pin is the main wear mechanism. The pin material is transferred as well as torn out deeply and then forms the layer over the surface. It seems that this type of wear may result from the mechanical properties of alloy 4 (Table 3). At the same time, the temperature in this area increases and oxides are formed. At a later stage of wear, due to heat and stresses, cracks appear, causing further release of these layers. Hard oxides cause micro-cutting. In this sample, there is a set of phenomena such as adhesive, abrasive wear, oxidation wear and fatigue [40,55].

### 3.5. Test Stand

**Figure 14:**
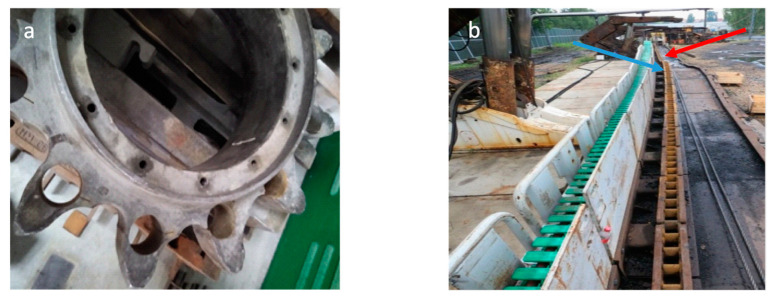
Elements of the longwall shearer haulage system: (**a**) drive wheel; (**b**) track.

After the laboratory test, an alloy on 350-toothed segments on the haulage system was selected. We chose alloy 0 for toothed segments and tested it on the test stand. A developed and constructed test stand was prepared on the ground. Most devices working in the mine were used to build test stands, for example, a longwall shearer or haulage system. After the full cycle of these tests, the before-selected and marked-toothed segments were analyzed. The main purpose of this work was to determine the weight loss of the examined toothed segments. Figure 14a shows the driving wheel of the longwall shearer, while Figure 14b shows the path of toothed bars. The blue arrow shows where the track rise, and the red arrow shows where the track turn. The drive wheel shown in Figure 14a was used in the typical mining cycle. The wheel could not work further.

**Figure 15:**
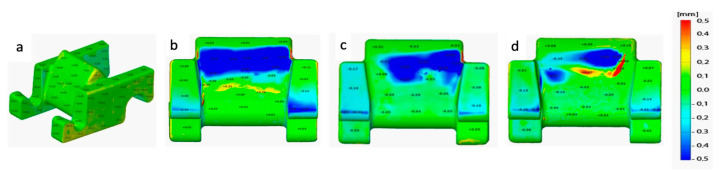
Test results of toothed segments: (**a**) toothed segments before the test; (**b**) uniform wear; (**c**) one-sided wear; (**d**) intensive one-sided wear.

3D scanning of the toothed segments was performed before the test as well as after the test on the prepared stand, which is shown in Figure 15. The results of the measurements showed differentiated ways to wear the tested toothed segments. The nature of the found dimensional changes resulting from toothed segments wear depending on the place in the track haulage system.

The toothed segment (Figure 15b) wear is evenly on the straight section. The segments placed on the bends had one-sided wear, as shown in Figure 15c. In the case of one of the tested toothed segments, the wear of the working surface was very intense, which was related to the impact of the drive wheel on a much smaller area toothed segment. The wear of this element is shown in Figure 15d. The deviation map shows the places of the highest wear of elements during operation under the test conditions.

Table 5 presents the results of the analysis of selected toothed segments after the tests were performed. The tested track was not straight on the track there was an arc both horizontally and vertically (Figure 14b), and in these areas, there was significant wear on the given toothed segment. A good example is the toothed segment D, located in the arc shown in Figure 14b. The wear of this toothed segment is definitely greater due to the nature of the toothed, as shown in Figure 15d. The lower wear of segment A results from the initial position of this element in the track. For the remaining toothed segments, the average wear was 34 g, while the weight loss of segment D was 2.7 times greater. For evenly worn toothed segments, each run causes average segment wear of 0.73 g.

The chemical composition of the wear products taken from the track was compared with the chemical composition of the toothed material (cast steel 0) and the drive wheel (Table 6). The results of the analysis show that the toothed segments are worn as a result of the processes taking place during the operation. This is also confirmed by the observation of the drive wheel. A significant presence in the examined samples of the drive wheel material would influence the elements level, mainly nickel.

## 4. Conclusions

The paper presents the results of wear tests of driving wheel material cooperating with selected alloys as a tooth. Understanding the wear mechanisms is a crucial element of tribological study. This knowledge can help solve engineering problems. The research was carried out at a laboratory station and a test stand. In the laboratory step, Amsler-A135 and TR-20 (pin-on-disc) tribometers were used for wear tests under dry friction conditions. During the research, eight alloys were tested, which formed friction pairs with carburized alloy 20H2N4A. Depending on the material used during tests, carburized surface samples’ wear mechanism was different. Adhesive wear, micro-cutting, powling, cracks, tracks of three body wear, plastic deformation and fatigue wear was reported after experiments. After the laboratory step, alloy 0 on 350 toothed segments on the haulage system has been selected. Toothed segments were subjected to tests on the test stand. The system was tested on the ground, and the results showed an increase in the service life of the track wheel in these conditions, which can contribute to reducing operating costs in the mining process. The main conclusions of the conducted research are presented below:

1. The tests performed using the pin-on-disc as well as the Amsler method were consistent for sample 0, and in both cases, the pair of 0/20H2N4A alloys showed the lowest wear under dry friction.
2. The wear of the carburized layer of the wheel material for all the tests carried out is associated with plowing, micro-cutting, adhesive wear, oxidation wear and fatigue. However, the share of individual wear mechanisms for the tested friction pairs using the pin-on-disk method is significantly different.
3. A relationship was found between the temperature and the wear of the drive wheel alloy; the higher temperature recorded during the Amsler test also causes greater wear. ADI grade 1–1 cast iron is an exception here.
4. In the case of pin-on-disc tests, an important mechanism causing the highest wear of drive wheel material (20H2N4A) was adhesive wear.
5. In the case of hardness and yield strength of the tested alloys, no relationship was found between these properties and the degree of wear.
6. Toothed segments in places of track guide bending wear more than 2.5 times faster than in straight sections. These areas become critical places that can potentially be a source of failure of the Longwall Shearer Haulage System.
7. The analysis confirmed that the drive wheel was not subjected to the process of wear, which extends the service life of the entire system.

Considering the nature of the longwall shearer’s haulage system, it can be expected that during the travels, it will be necessary to adjust the drive track to the coal seams many times. The research carried out in this work confirms that it is possible to select the appropriate material for toothed critical areas (Figure 15d). In this case, it would be necessary to divide the toothed segments into two groups. The issue that needs to be resolved is the logistical ability to separate these two groups of toothed segments in production conditions. The magnitude of the impact of these changes on the wear of the drive wheel should be checked. Further research has required the reasons for raising the friction coefficient for sample 0 during pin-on-disc tests.

## Figures and Tables

**Table 1:** Chemical compositions of the tested alloys.

Alloy		Chemical Composition Analysis Results; wt%										
Material	Symbol	C	SI	MN	P	S	CR	NI	MO	MG	V	B
Steel—drive wheel—surface	**20H2N4A**	0.59	0.22	0.37	0.009	0.012	1.34	3.14	0.026	-	-	-
Steel—drive wheel—core	**20H2N4A**	0.17	0.22	0.37	0.008	0.010	1.32	3.08	0.025	-	-	-
Cast steel—toothed segment	**0**	0.24	0.80	0.97	0.014	0.004	0.86	1.18	0.16	-	-	-
ADI—toothed segment	**1**	3.6	2.45	0.32	0.040	0.018	-	1.9	-	0.65	-	-
Cast steel—toothed segment	**2**	0.25	0.37	1.41	0.023	0.023	1.54	0.05	0.44	-	-	0.007
Hadfield cast steel—toothed segment	**3**	0.90	1.10	12.61	0.030	0.009	1.20	0.36	0.44			
Cast steel—toothed segment	**4**	0.47	1.76	0.72	0.017	0.012	0.94	1.740	0.570	-	0.140	-
Cast steel—toothed segment	**5**	0.23	0.42	1.08	0.02	0.005	0.92	1.02	0.61	-	0.250	-

**Table 2:** Symbols and heat treatment parameters tested alloys.

Symbol	Heat Treatment Process Parameters
**20H2N4A**	− condition delivered
**0**	− normalizing: 930 °C/60 min/with a furnace − quenching in water: 920 °C/60 min/water − tempering: 250 °C/60 min/air
**1–1**	− austenitization 900 °C/120 min − quenching in salt 270 °C/180 min
**1–2**	− austenityzacja 900 °C/120 min − quenching in salt 330 °C/150 min
**1–3**	− austenityzacja 900 °C/120 min − quenching in salt 375 °C/150 min
**2**	− quenching in water: 950 °C/60 min/water − tempering: 250 °C/60 min/air
**3**	− quenching in water 1070 °C/ 90 min/water
**4**	− normalizing: 930 °C/60 min/with a furnace − quenching in oil: 890 °C/60 min/oil − tempering: 310 °C/60 min/air + 310 °C/60 min/air
**5**	− condition delivered

**Table 3:** Mechanical properties of the analyzed alloys.

Symbol	0	1–1	1–2	1–3	2	3	4	5
TS [MPa]	1070	1480	1170	950	1514	1040	1683	1270
YS [MPa]	970	1130	830	580	1415	410	1537	820
A [%]	9.5	2.4	5.6	8.2	8.4	4	2.5	10

**Table 4:** EDS analysis results.

Elements	Weight, %					
	Spot 1	Spot 2	Spot 3	Spot 4	Spot 5 *	Spot 6
C	8.2	14.5	10.0	10.5	9.6	8.2
Si	0.3	0.3	-	0.4	-	-
Cr	1.0	3.0	0.4	1.1	1.0	0.5
Ni	2.9	0.6	-	3.0	2.5	0.9
Fe	87.5	81.5	89.3	84.9	81.6	90.4

* Spot area 5 analysis revealed 5.3% oxygen content.

**Table 5:** Weight loss of selected toothed segments.

Mark		A	B	C	D	E	F	G	H	J
Weigh loss	[g]	11	37	27	92	38	36	38	27	36

**Table 6:** Chemical composition of the tested samples.

Nr	Si	Mn	Cr	Mo	Ni
	Chemical Composition; wt.%				
1	0.858	1.023	0.914	0.161	1.204
2	0.896	0.982	0.767	0.147	1.015
